# Association between aspirin‐induced hemoglobin decline and outcome after acute ischemic stroke in G6PD‐deficient patients

**DOI:** 10.1111/cns.13711

**Published:** 2021-08-08

**Authors:** Yicong Chen, Jianle Li, Zilin Ou, Yusheng Zhang, Zhijian Liang, Weisheng Deng, Weixian Huang, Fubing Ouyang, Jian Yu, Shihui Xing, Jinsheng Zeng

**Affiliations:** ^1^ Department of Neurology, The First Affiliated Hospital, Sun Yat‐sen University Guangdong Provincial Key Laboratory for Diagnosis and Treatment of Major Neurological Diseases, National Key Clinical Department and Key Discipline of Neurology Guangzhou China; ^2^ Department of Neurology The First Affiliated Hospital of Jinan University Guangzhou China; ^3^ Department of Neurology The First Affiliated Hospital of Guangxi Medical University Nanning China; ^4^ Department of Neurology Meizhou People’s Hospital Meizhou China; ^5^ Department of Neurology and Stroke Center, The First Affiliated Hospital, Sun Yat‐sen University Guangdong Provincial Key Laboratory for Diagnosis and Treatment of Major Neurological Diseases, National Key Clinical Department and Key Discipline of Neurology Guangzhou China

**Keywords:** aspirin, G6PD deficiency, hemoglobin, outcome, stroke

## Abstract

**Aims:**

The risk of hemoglobin decline induced by low‐dose aspirin in glucose‐6‐phosphate dehydrogenase (G6PD) deficiency remains unknown, and its influence on stroke outcome remains to be investigated. This study aimed to evaluate the effect of G6PD deficiency on hemoglobin level during aspirin treatment and its association with outcome after acute ischemic stroke.

**Methods:**

In total, 279 patients (40 G6PD‐deficient and 239 G6PD‐normal) with acute ischemic stroke treated with aspirin 100 mg/day from a cohort study were examined. The primary safety endpoint was a hemoglobin decline ≥25 g/L or 25% from baseline within 14 days after aspirin treatment. Poor outcomes were defined as a modified Rankin Scale score ≥2 at 3 months. The *χ* ^2^ test was used to compare stroke outcomes, and multivariate logistic regression analyses were performed to analyze the association between hemoglobin level and outcomes.

**Results:**

The G6PD‐deficient group had lower baseline hemoglobin and tended to develop comorbid pulmonary infection more frequently (*p* < 0.05). The proportion of patients with hemoglobin decline ≥25 g/L or 25% from baseline (15.0% vs. 3.3%; *p* = 0.006) and anemia (30.0% vs. 14.6%; *p* = 0.016) after aspirin treatment was higher in the G6PD‐deficient group, which was accompanied by a more significant bilirubin increase. The rate of poor functional outcomes at 3 months after acute ischemic stroke was higher in the G6PD‐deficient group (Risk ratio = 1.31 [95% confidence interval (CI) = 1.10–1.56]; *p* = 0.017). Confounder‐adjusted analysis showed that lower hemoglobin levels (odds ratio = 0.98 [95% CI = 0.96–0.99]; adjusted *p* = 0.009) increased the risk of poor functional outcomes.

**Conclusion:**

Hemoglobin decrease with bilirubin increase after aspirin treatment in patients with G6PD deficiency suggests hemolysis, which may influence stroke prognosis. The risk of hemoglobin decline should be carefully monitored in G6PD‐deficient patients with ischemic stroke taking aspirin.

## Graphical abstract

Hemoglobin decrease with bilirubin increase after aspirin treatment in patients with G6PD deficiency suggests hemolysis, which may influence stroke prognosis. The risk of hemoglobin decline should be carefully monitored in G6PD‐deficient stroke patients taking aspirin.

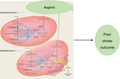

## 1 INTRODUCTION

Glucose‐6‐phosphate dehydrogenase (G6PD) deficiency is the most common human enzyme defect, affecting more than 500 million individuals worldwide.^1^ In southern China, the prevalence of G6PD deficiency reportedly varies from 4% to 10%.^2^ Aspirin is the most extensively used antiplatelet agent for secondary stroke prevention,^2^ but its safety in patients with G6PD deficiency is controversial.^1^, ^3^, ^4^, ^5^, ^6^, ^7^, ^8^, ^9^, ^10^ Our previous study revealed a higher risk of moderate‐to‐severe bleeding after aspirin used in patients with G6PD deficiency,^3^ which might lead to a decline in hemoglobin. However, the hemoglobin decline was accompanied by a significant bilirubin increase after aspirin treatment in the G6PD‐deficient patients, suggesting hemolysis. Aspirin‐induced acute symptomatic hemolysis associated with G6PD deficiency has been reported since the 1960s,^7^, ^8^, ^9^, ^10^, ^11^ but a recent series of case reports suggested that a low‐dose aspirin (100 mg/daily) was adequately safe for these patients.^6^, ^12^, ^13^, ^14^ The risk of aspirin‐induced asymptomatic hemolysis in those with G6PD deficiency remains unknown. Pulmonary infection is a common comorbidity after stroke,^2^ which may increase the risk of developing hemolysis in those with G6PD deficiency using aspirin, even if low dose (100 mg/daily).^15^, ^16^ Compared with that caused by digestion of fava beans, drug‐induced hemolysis in G6PD deficiency is often self‐limiting or asymptomatic, which has not received enough attention.^6^, ^7^

Recently, we found worse stroke outcomes in patients with G6PD deficiency than in those without,^17^ but the mechanism is unclear. Low hemoglobin levels or anemia has been demonstrated to be associated with poor stroke outcomes.^18^, ^19^, ^20^, ^21^, ^22^, ^23^ Whether hemoglobin decline induced by aspirin in G6PD‐deficient patients influences stroke outcomes remains unknown. In this study, we evaluated the risk of hemoglobin decline and bilirubin increase within 14 days after low‐dose aspirin in patients with different G6PD statuses and further investigated its association with functional outcomes at 3 months after stroke, to clarify the safety of aspirin use in those with G6PD deficiency.

## 2 METHODS

### 2.1 Study design and population

This study was a post hoc analysis of a prospective, multicenter, and hospital‐based cohort study that enrolled patients with ischemic stroke who were admitted within 7 days from symptom onset at the four representative stroke centers in South China between March 2015 and November 2017. The participants eligible for this study (1) were aged ≥18 years; (2) had an ischemic stroke confirmed via computed tomography or magnetic resonance imaging; (3) were on aspirin 100 mg/daily as monotherapy or as part of dual antiplatelet therapy with clopidogrel; and (4) had available data on hemoglobin and serum bilirubin before aspirin treatment and within 14 days after aspirin treatment. The exclusion criteria were (1) a modified Rankin Scale (mRS) score of ≥2 before stroke onset; (2) a baseline hemoglobin level <90 g/L; (3) comorbidity with hemolytic diseases, including thalassemia; (4) with intravenous thrombolysis or mechanical thrombectomy; (5) aspirin ingestion 1 month before enrollment; and (6) an expected survival duration ≤3 months. Aspirin was administered on hospital day 1. All patients were requested to refrain from additional medications contraindicated for G6PD deficiency, including traditional Chinese medicines.^15^ The study was approved by the ethics committee of the four study centers (the First Affiliated Hospital of Sun Yat‐sen University, the First Affiliated Hospital of Jinan University, the First Affiliated Hospital of Guangxi Medical University, and Meizhou People's Hospital). Informed consent was obtained from all participants or their representatives.

### 2.2 Evaluation of G6PD levels

The G6PD level was tested on the second day after admission. G6PD deficiency was defined as G6PD enzyme activity <1300 U/L or as a G6PD/6‐phosphogluconate dehydrogenase ratio <1.0, which were then identified by G6PD exon sequencing. The ratio was determined using a quantitative assay of the rate of reduced nicotinamide adenine dinucleotide phosphate hydrogen production from nicotinamide adenine dinucleotide phosphate.^15^ The participants were accordingly divided into the G6PD‐deficient group or the G6PD‐normal group based on their G6PD level.

### 2.3 Data collection

Baseline data, including demographics, clinical features, vascular risk factors, National Institutes of Health Stroke Scale (NIHSS) scores, hematologic parameters, comorbidities, treatments, and duration of hospitalization, were collected. Vascular risk factors included the history of ischemic stroke, transient ischemic attack (TIA), hypertension, diabetes mellitus, hyperlipidemia, coronary heart disease, atrial fibrillation, smoking, and drinking.

The primary safety endpoint was the rate of hemoglobin decline ≥25 g/L or 25% from baseline^24^ within 14 days after aspirin treatment. Anemia was defined as a hemoglobin level <120 g/L in men and <110 g/L in women according to the Chinese criteria.^25^ The difference in serum bilirubin between the pre‐ and post‐aspirin period, defined as an increase of ≥2.5 mmol/L or 20% from baseline, was also compared. The hemoglobin and bilirubin levels were examined before and within 14 days after aspirin treatment; the changes were calculated by subtracting the pre‐aspirin value from the post‐aspirin value. Bleeding was defined according to the Global Utilization of Streptokinase and Tissue Plasminogen Activator for Occluded Coronary Arteries (GUSTO) definition.^26^

Functional outcomes were determined according to the mRS score at 3 months, which was evaluated by researchers blinded to the G6PD status. The mRS score ranges from 0 to 6, with 0 indicating no symptoms; 1, no clinically significant disability; 2, slight disability (the patient is able to look after their own affairs without assistance but is unable to carry out all previous activities); 3, moderate disability (the patient requires some help but is able to walk unassisted); 4, moderately severe disability (the patient is unable to attend to bodily needs without assistance and unable to walk unassisted); 5, severe disability (the patient requires constant nursing care and attention); and 6, death.^27^ Functional outcomes were defined as “well” if patients had a mRS score of <2 and “poor” if their mRS score was ≥2.^24^ The participants were followed up at 3 months through face‐to‐face visits at the local hospital or via telephonic conversation for those who were unable to or refused to return to the hospital.

### 2.4 Statistical analysis

Kolmogorov‐Smirnov formal test was used to assess the data distribution. Categorical variables were presented as percentages, while continuous variables were presented as medians with interquartile ranges because of their non‐normal distribution. The *χ* ^2^ test and Wilcoxon rank sum test were performed for between‐group comparisons of categorical variables and continuous variables, respectively. Differences in outcomes were assessed using the *χ* ^2^ test and risk ratios (RR) with 95% confidence interval (CI) were reported. Risk factors associated with hemoglobin decline and functional outcomes at 3 months were analyzed using univariate and multivariate logistic regression analyses. Odds ratios (OR) and 95% CIs were calculated. All statistical analyses were performed using SPSS 23.0 (SPSS Inc., Chicago, IL, USA). A *p* value < 0.05 was considered significant.

## 3 RESULTS

### 3.1 Patient characteristics

**FIGURE 1:**
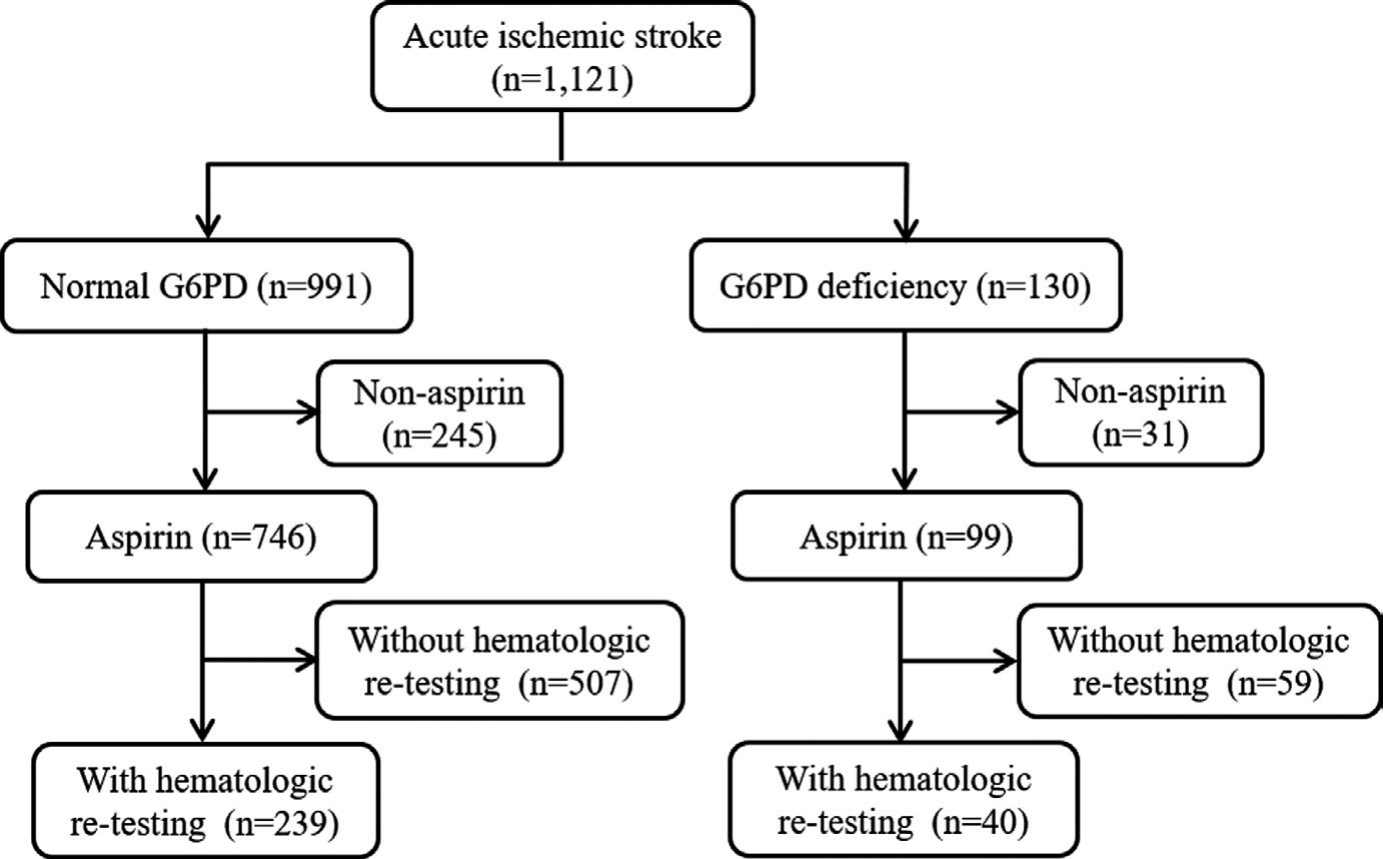
Flowchart of patients included. G6PD, glucose‐6‐phosphate dehydrogenase

Overall, 1,121 patients with acute ischemic stroke were screened; 130 (11.6%) were identified as G6PD‐deficient and 991 (88.4%) as G6PD‐normal (Figure 1). Aspirin was prescribed for 99 G6PD‐deficient and 746 G6PD‐normal patients. Finally, 279 patients (40 G6PD‐deficient patients and 239 G6PD‐normal patients) who underwent 14‐day re‐testing of hematologic parameters were included in the analysis. The median patient age was 64 years, and 188 patients (67.4%) were men. Compared with the G6PD‐normal group, the G6PD‐deficient group had a lower baseline hemoglobin level (median: 134.5 vs. 140.5 g/L; *p* = 0.008) and a higher frequency of comorbid pulmonary infection during hospitalization (32.5% vs. 15.9%; *p* = 0.012) (Table 1). The two groups were balanced with respect to age, sex, history of stroke/TIA, hypertension, diabetes mellitus, hyperlipidemia, coronary heart disease, atrial fibrillation, smoking, drinking, baseline NIHSS score, anemia, serum bilirubin, the proportion of dual antiplatelet therapy, and hospitalization duration (all *p* > 0.05).

**TABLE 1:** Patient characteristics

Characteristic	G6PD‐deficient	G6PD‐normal	*p* value
	(*n* = 40)	(*n* = 239)	
Age, Median (IQR)	68 (56, 77)	64 (55, 75)	0.499
Male, % (*n*/*N*)	57.5 (23/40)	69.0 (165/239)	0.150
Medical history, % (*n*/*N*)			
Previous stroke/TIA	17.5 (7/40)	13.8 (33/239)	0.537
Hypertension	80.0 (32/40)	75.7 (181/239)	0.557
Diabetes	27.5 (11/40)	30.5 (73/239)	0.698
Hyperlipidemia	25.0 (10/40)	24.7 (59/239)	0.966
Coronary heart disease	7.5 (3/40)	12.6 (30/239)	0.515
Atrial fibrillation	5.0 (2/40)	6.7 (16/239)	0.955
Smoking, % (*n*/*N*)	32.5 (13/40)	40.2 (96/239)	0.358
Drinking, % (*n*/*N*)	15.0 (6/40)	19.2 (46/239)	0.523
NIHSS score, Median (IQR)	7 (4, 10)	5 (3, 10)	0.111
Baseline hemoglobin, Median (IQR), g/L	134.5 (121.9, 141.3)	140.5 (130.0, 152.0)	0.008
Anemia, % (*n*/*N*)	15.0 (6/40)	7.9 (19/239)	0.252
Baseline serum bilirubin, Median (IQR), μmol/L	14.8 (9.6, 22.2)	13.4 (9.0, 18.6)	0.450
Pulmonary infection, % (*n*/*N*)	32.5 (13/40)	15.9 (38/239)	0.012
Dual antiplatelet, % (*n*/*N*)	30.0 (12/40)	37.7 (90/239)	0.352
Hospitalization duration, Median (IQR), days	13 (10, 17)	12 (9, 15)	0.483

Abbreviations: G6PD, glucose‐6‐phosphate dehydrogenase; IQR, interquartile range; NIHSS, National Institute of Health Stroke Scale; TIA, transient ischemic attack.

### 3.2 Hemoglobin levels

The rate of hemoglobin decline ≥25 g/L or 25% from baseline within 14 days after aspirin treatment was notably higher in the G6PD‐deficient group than in the G6PD‐normal group (15.0% vs. 3.3%; *p* = 0.006) (Table 2). The rate of anemia after using aspirin was also higher in this group (30.0% vs. 14.6%; *p* = 0.016). Patients with G6PD deficiency also showed a more obvious increase in serum bilirubin after aspirin treatment (Table 2). The proportion of patients with bilirubin increase of ≥2.5 μmol/L or 20% from baseline was significantly higher in the G6PD‐deficient group than in the G6PD‐normal group (73.3% vs. 41.4%; *p* < 0.001). When the patients with dual antiplatelets were excluded, the G6PD‐deficient group using aspirin alone also demonstrated high risks in hemoglobin decline, anemia, and bilirubin increase (Table S1). No symptomatic hemolytic crisis was observed in either group after using aspirin. The risk of moderate‐to‐severe bleeding tended to be higher in those with G6PD deficiency (OR = 12.11 [95% CI = 1.07–137.05]; *p* = 0.058; Table S2).

**TABLE 2:** Hemoglobin decline and bilirubin increase within 14 days after aspirin treatment

Parameters	G6PD‐deficient	G6PD‐normal	*p* value
	(*N* = 40)	(*N* = 239)	
Hemoglobin decrease ≥25 g/L or 25%, % (*n*/*N*)	15.0 (6/40)	3.3 (8/239)	0.006
Anemia, % (*n*/*N*)	30.0 (12/40)	14.6 (35/239)	0.016
Bilirubin increase ≥2.5 mmol/L or 20%, % (*n*/*N*)	73.3 (29/40)	41.4 (99/239)	<0.001

Abbreviation: G6PD, glucose‐6‐phosphate dehydrogenase.

Univariate logistic regression analysis showed that hemoglobin decline was associated with G6PD deficiency (unadjusted *p* = 0.004) and pulmonary infection (unadjusted *p* = 0.004) (Table 3). G6PD deficiency (OR = 4.86 [95% CI = 1.47–16.09]; adjusted *p* = 0.010) and pulmonary infection (OR = 5.09 [95% CI = 1.57–16.52]; adjusted *p* = 0.007) were confirmed to be risk factors for hemoglobin decline in the multivariate logistic analysis. Age (OR = 0.94 [95% CI = 0.89–0.99]; adjusted *p* = 0.019) was found to be a protective factor after adjusting for confounding factors such as sex, NIHSS score, and bleeding after aspirin treatment.

**TABLE 3:** Univariate and multivariate logistic regression analysis of risk factors for hemoglobin decline after aspirin treatment

Variables	Univariate		Multivariate	
	Odds ratio (95% CI)	*p* value	Odds ratio (95% CI)	*p* value
Age	0.96 (0.91, 1.00)	0.058	0.94 (0.89, 0.99)	0.019
Female	0.55 (0.15, 2.02)	0.366	…	0.431
G6PD deficiency	5.10 (1.67, 15.59)	0.004	4.86 (1.47, 16.09)	0.010
Pulmonary Infection	5.02 (1.68, 15.04)	0.004	5.09 (1.57, 16.52)	0.007
NIHSS score	1.01 (0.92, 1.11)	0.836	…	0.386
Bleeding after aspirin treatment	1.01 (0.99, 1.02)	0.374	…	0.391

Note

Models were built using stepwise regression with variables entering into the model at the 0.10 significance level and removed at the 0.05 significance level.

Abbreviations: CI, confidence interval; G6PD, glucose‐6‐phosphate dehydrogenase; NIHSS, National Institute of Health Stroke Scale.

Considering the impact of pulmonary infection on hemoglobin decline, we further evaluated the change in hemoglobin levels among patients without pulmonary infection. In those without pulmonary infection, the rate of hemoglobin decline ≥25 g/L or 25% from baseline was also higher in the G6PD‐deficient group than in the G6PD‐normal group (11.1% vs. 2.0%; *p* = 0.038) (Table S3).

### 3.3 Functional outcomes

Patients with G6PD deficiency (82.5%) demonstrated poorer functional outcomes at 3 months after ischemic stroke than those without G6PD deficiency (63.2%) (RR = 1.31 [95% CI = 1.10–1.56]; *p* = 0.017). Moreover, patients developing anemia within 14 days after aspirin treatment also had worse functional outcomes at 3 months (80.9% vs. 62.9%; RR = 1.29 [95% CI = 1.08–1.52]; *p* = 0.018).

A lower hemoglobin level post‐aspirin treatment was found to independently increase the risk of poor functional outcomes (OR = 0.98 [95% CI = 0.96–0.99]; adjusted *p* = 0.009) (Table 4), after adjusting for age, sex, previous stroke/TIA, hypertension, diabetes, NIHSS score, and pulmonary infection. Further, a higher NIHSS score at baseline was also associated with poor stroke outcomes (OR = 1.44 [95% CI = 1.29–1.60]; adjusted *p* = 0.001).

**TABLE 4:** Univariate and multivariate logistic regression analyses of variables for poor functional outcomes at 3 months

Variables	Univariate		Multivariate	
	Odds ratio (95% CI)	Unadjusted *p*	Odds ratio (95% CI)	Adjusted *p*
Age	1.02 (1.00, 1.04)	0.101	…	0.317
Sex	1.32 (0.74, 2.36)	0.347	…	0.984
Previous stroke/TIA	0.88 (0.42, 1.88)	0.881	…	0.296
Hypertension	1.12 (0.64, 2.15)	0.607	…	0.313
Diabetes	0.71 (0.40, 1.25)	0.231	…	0.373
NIHSS score	1.41 (1.28, 1.59)	<0.001	1.44 (1.29, 1.60)	<0.001
Pulmonary infection	4.64 (1.74, 12.39)	0.002	…	0.249
Hemoglobin post‐aspirin	0.98 (0.97, 0.99)	0.020	0.98 (0.96, 0.99)	0.009

Note

Models were built using stepwise regression with variables entering into the model at the 0.10 significance level and removed at the 0.05 significance level.

Abbreviations: CI, confidence interval; NIHSS, National Institutes of Health Stroke Scale; TIA, transient ischemic attack.

## 4 DISCUSSION

The risk of hemoglobin decline in patients with G6PD deficiency and its influence on stroke outcomes remain unknown. In this study, patients with ischemic stroke and G6PD deficiency demonstrated a higher risk of hemoglobin decline and anemia. The decline in hemoglobin was accompanied by a significant increase in bilirubin at an early stage after aspirin treatment, which strongly suggested asymptomatic hemolysis. Hemoglobin decline was decidedly associated with G6PD deficiency, and it increased the risk of poor outcomes at 3 months after stroke. The risk of hemoglobin decline resulting from asymptomatic hemolysis with aspirin treatment should be paid attention to in G6PD‐deficient patients.

Our previous study found that G6PD‐deficient patients receiving aspirin had a higher risk of moderate‐to‐severe bleeding.^3^ However, hemoglobin decline was accompanied by a significant bilirubin increase, which suggested the occurrence of asymptomatic hemolysis. Besides, the numbers of moderate‐to‐severe bleeding events were small in this analysis, and they did not significantly contribute to hemoglobin decline (Table 3). Aspirin is used as a hemolytic agent for individuals with G6PD deficiency owing to its oxidizing property, but the associated risks remain controversial.^4^, ^5^, ^6^, ^7^, ^8^, ^9^, ^10^, ^11^, ^12^, ^13^, ^14^, ^28^, ^29^, ^30^ Aspirin‐related acute hemolytic crisis is not common, and the risk of asymptomatic hemolysis does not draw enough attention. Aspirin‐related acute hemolysis in a patient with G6PD deficiency was first reported in 1960. The patient was a 19‐year‐old soldier who presented with fever and abdominal pain and developed acute hemolysis 1 day after receiving 1800 mg aspirin.^9^ Since then, cumulative cases have shown that high‐dose aspirin (>100 mg/daily) causes hemolytic crisis in patients with fever or rheumatic diseases and G6PD deficiency.^7^, ^8^, ^10^, ^11^ However, a good safety profile was recently reported in nine G6PD‐deficient patients who received aspirin at 100 mg/daily.^6^, ^12^, ^13^, ^14^, ^29^ Drug‐induced hemolysis in G6PD deficiency is generally self‐limiting or asymptomatic^6^, ^7^; therefore, evaluation of asymptomatic hemolysis is important. Although no hemolytic crises were observed in the above nine patients, two patients showed a significant decrease in hemoglobin levels (132 to 119 g/L and 148 to 128 g/L) several days following aspirin use.^14^, ^29^ This suggested the occurrence of asymptomatic hemolysis induced by low‐dose aspirin.

Minimal changes in hemoglobin concentration were observed following a 4‐day trial of aspirin (50 mg/kg/daily) in 6 patients with G6PD deficiency.^4^ Another study, with a longer follow‐up of 3 months, did not find a significant difference in complete blood count and serum bilirubin concentration between the pre‐aspirin and post‐aspirin values (250 mg/daily) in 44 ischemic heart disease patients with G6PD deficiency.^5^ However, the population in the first study included only 6 patients and the observation duration was 4 days; the second study had a much longer follow‐up of up to 3 months. Both studies, which exclusively enrolled G6PD‐deficient patients, did not compare the risk of asymptomatic hemolysis with that in G6PD‐normal individuals. Hemolysis generally occurred several days (1–10 days) after receiving aspirin.^7^, ^9^, ^10^, ^11^ Therefore, in this study, we chose the range of within 14 days for hematologic re‐testing following aspirin treatment. In vivo studies have found that aspirin could shorten red blood cell survival.^31^, ^32^ Aspirin itself has an acetylation effect on the erythrocyte membrane.^32^ Oxidative damage mediated via the aspirin metabolite gentisic acid was proposed as a mechanism for aspirin‐induced hemolysis.^32^, ^33^ Our study confirmed the risk of asymptomatic hemolysis following low‐dose aspirin in G6PD deficiency, indicating that these patients should be carefully monitored when prescribed with aspirin.

Infection is a risk factor for hemolysis in G6PD deficiency.^15^, ^16^ Hepatitis viruses A and B, cytomegalovirus,^34^ pneumonia,^35^ and typhoid fever are all notable causes. In our study, aspirin‐induced hemoglobin decline and its influence on stroke outcomes were independent of pulmonary infection. It is likely that the effects of G6PD deficiency on the associated hemoglobin decline and stroke outcome would be more common in men because G6PD deficiency is X‐linked. However, participants with stroke consisted largely of men, which might explain why this effect was not prominent in our study.

An older age seemed to be a protective factor against aspirin‐induced asymptomatic hemolysis in G6PD‐deficient patients. To date, all reported cases of aspirin‐related hemolysis occurring in G6PD deficiency were on individuals younger than 30 years old, including children.^7^, ^8^, ^9^, ^10^, ^11^ This might be one of the reasons that limited attention has been paid to the risk of aspirin‐related hemolysis related to G6PD deficiency in patients with stroke, as these individuals are usually aged 45 years or older. As for the mechanisms, only one study comparing the G6PD activity between aging and young mice was found.^36^ It observed that age‐dependent differences in regional brain G6PD activities, ranging from 2.0‐ to 7.0‐fold across brain regions higher in aging mice than young mice. The activity of G6PD in red blood cells demonstrated a “U”‐shaped curve with highest activities below 200 days of age, lowest activities between 200 and 400 days of age, returning to higher activities after 400 days of age in female heterozygous (+/def) G6PD‐deficient mice. The increase in G6PD activity with age may in part constitute an attempt to compensate for antioxidative enzymes such as glutathione reductase and SOD, which decline with age. The protective effect of an older age might partially explain the mild hemolytic symptom in G6PD‐deficient patients with ischemic stroke, as compared with that in younger individuals, but the exact mechanisms need further investigation.

Our recent study suggested that G6PD‐deficient patients had worse functional outcomes at 3 months after ischemic stroke,^17^ which was confirmed in this study. The severity of stroke, as indicated by a higher NIHSS, predicts poor functional outcomes. In our study, multivariable analyses did show that a NIHSS score significantly influent outcome at 3 months after ischemic stroke (Table 4). We also found that a low hemoglobin level was associated with a poor stroke prognosis, and the lower hemoglobin level after using aspirin was associated with G6PD deficiency. A lower hemoglobin level or anemia significantly increases the risk of poor functional outcomes, mortality, and stroke recurrence in patients with stroke.^18^, ^20^, ^21^, ^22^, ^23^ By lowering the oxygen‐carrying capacity of the blood, anemia may worsen ischemia and subsequently hypoxia within the penumbral lesions in patients with ischemic stroke, which worsen neurologic deficits and influenced stroke outcomes.^23^ The lack of oxygen and energy supply has a profound effect on the cerebral vascular regulation over time.^37^ Lower hemoglobin and hematocrit values were associated with higher baseline parenchymal cerebral blood flow (CBF) and slower decline in parenchymal CBF over time.^38^ The increase in CBF augments tissue oxygen delivery but at the cost of a potential increase in the number of cerebral embolic events.^37^ Such emboli may contribute to the observed increase in the incidence of ischemic injury. In addition, anemia may trigger the inflammation response, and some inflammatory markers associated with stroke prognosis, such as C‐reactive protein and tumor necrosis factor‐α, have been found to be increased in patients with anemia.^39^, ^40^ A significant decline in hemoglobin levels of G6PD‐deficient patients treated with aspirin may produce an unfavorable effect on functional outcomes; thus, this special group should be prescribed aspirin with caution.

This study has some limitations. As this was an observational study, some patients failing to have complete 14‐day re‐testing blood parameters were excluded from final analyses. The reasons included lack of real baseline data (using antiplatelet before blood examination), rejection of re‐testing, and uncomplete re‐testing data (with hemoglobin level, but without serum bilirubin). Some of the confounding factors, such as comorbidities and medications, can also probably lead to hemoglobin decline. However, we required all patients to refrain from additional medications contraindicated for G6PD deficiency. Another limitation is that the effect of G6PD activity on the risk of aspirin‐induced hemolysis could not be analyzed in our study, because we only preliminarily screened for G6PD deficiency. Moreover, the study did not have a large sample size, and approximately 36.5% of patients with aspirin as part of their dual antiplatelet therapy with clopidogrel were included. After excluding those with dual antiplatelet, higher risk of hemoglobin decline and bilirubin increase were also found in G6PD‐deficient patients using aspirin alone. There were only 7 patients with G6PD deficiency and 60 patients with normal G6PD taking clopidogrel alone with complete hematological data in our analyses. The decrease in hemoglobin and increase in serum bilirubin were not significantly different between patients with G6PD deficiency or not. Not any clinical hemolysis crisis was observed in G6PD‐deficient patients with clopidogrel in our study, nor be reported in the other literature. However, we could not make a definite conclusion regarding the safety of clopidogrel based on our data because of the limited patient sample. To overcome these limitations, we are conducting a randomized controlled trial comparing the safety and efficacy of aspirin (100 mg/daily) versus clopidogrel (75 mg/daily) in patients with ischemic stroke and G6PD deficiency with periodic re‐examination of hematologic parameters, in order to further elucidate the safety and the efficacy of low‐dose aspirin in patients with G6PD deficiency (https://clinicaltrials.gov/ct2/show/NCT04088513).

## 5 CONCLUSION

Patients with G6PD deficiency might develop hemoglobin decline as a result of asymptomatic hemolysis in the early stage after aspirin treatment, and this may influence functional outcomes after ischemic stroke. Thus, the G6PD activity should be screened when prescribing aspirin to patients with stroke, and the hemoglobin levels should be carefully monitored in those with G6PD deficiency.

## CONFLICTS OF INTEREST

The authors declare that there is no conflict of interest that could be perceived as prejudicing the impartiality of the research reported.

## Supporting information

Table S1
Table S2
Table S3

## Data Availability

The data that support the findings of this study are available from the corresponding author upon reasonable request.
