# Supplementary material for: Association between aspirin‐induced hemoglobin decline and outcome after acute ischemic stroke in G6PD‐deficient patients
Source: CNS Neurosci Ther. 2021 Aug 8;27(10):1206–13. doi: 10.1111/cns.13711 (PMC8446213; doi:10.1111/cns.13711)
Supplement: Supplementary file 1 — Table S1 Table S2 Table S3 [file CNS-27-1206-s001.docx]

**Supplemental Data**

**Supplemental Table e-1** **Hemoglobin decline and bilirubin increase within 14 days after treated with aspirin alone**

|  | **G6PD-deficient** | **G6PD-normal** | **P value** |
| --- | --- | --- | --- |
| **Hemoglobin decrease ≥25 g/L or 25%, % (n/N)** | 17.9 (5/28) | 2.7 (4/149) | 0.004 |
| **Anemia, % (n/N)** | 35.7 (10/28) | 14.1 (21/149) | 0.013 |
| **Bilirubin increase ≥2.5 mmol/L or 20%, % (n/N)** | 82.1 (23/28) | 43.5 (67/149) | 0.007 |

G6PD = glucose-6-phosphate dehydrogenase.

**Supplemental Table e-2 Risk of bleeding after aspirin treatment**

| **Bleeding*** | **G6PD deficiency  (N = 38)** | |  | **Normal G6PD  (N = 219)** | | **Odds ratio  (95% CI)** | **P value** |
| --- | --- | --- | --- | --- | --- | --- | --- |
|  | Event, n | Event rate, % |  | Event, n | Event rate, % |  |  |
| **Any bleeding** | 3 | 7.9 |  | 7 | 3.2 | 2.60 (0.64, 10.52) | 0.353 |
| **Moderate-severe bleeding** | 2 | 5.3 |  | 1 | 0.5 | 12.11 (1.07, 137.05) | 0.058 |

*Bleeding events were defined according to the Global Utilization of Streptokinase and Tissue Plasminogen Activator for Occluded Coronary Arteries criteria as follows: severe bleeding was defined as fatal or intracranial hemorrhage or other hemorrhage causing hemodynamic compromise that required blood or fluid replacement, inotropic support, or surgical intervention; moderate bleeding as bleeding that required transfusion of blood but did not lead to hemodynamic compromise requiring intervention; and mild bleeding as bleeding not requiring transfusion and not causing hemodynamic compromise (e.g., subcutaneous bleeding, mild hematomas, and oozing from puncture sites, not including gingival bleeding).

CI = confidence interval; G6PD = glucose-6-phosphate dehydrogenase.

**Supplemental Table e-3. Hemoglobin decline and bilirubin increase within 14 days after aspirin treatment in patients without pulmonary infection**

|  | **G6PD-deficient** | **G6PD-normal** | **P value** |
| --- | --- | --- | --- |
| **Hemoglobin decline of ≥25 g/L or 25%, (n/N)** | 11.1 (3/27) | 2.0 (4/201) | 0.038 |
| **Anemia, % (n/N)** | 22.2 (6/27) | 11.9 (24/201) | 0.238 |
| **Bilirubin increase ≥2.5 mmol/L or 20%, % (n/N)** | 51.9 (14/27) | 37.8 (76/201) | 0.161 |

G6PD = glucose-6-phosphate dehydrogenase.
